# Perceptions, Attitudes, and Expectations Toward Primary Health Care Service Provision and Their Association with Service Utilization in Subdistrict Health Promoting Hospitals in Northeastern Thailand: A Cross-Sectional Study

**DOI:** 10.3390/healthcare14162632

**Published:** 2026-08-20

**Authors:** Kanokporn Somporn, Niwat Songsin, Sarayut Chusuton, Sittichai Singsu, Adithep Phranphithak

**Affiliations:** 1 Department of Community Public Health, College of Allied Health Sciences, Suan Sunandha Rajabhat University, Bangkok 10300, Thailand; kanokporn.so@ssru.ac.th (K.S.); sarayut.ch@ssru.ac.th (S.C.); 2 Department of Occupational Health and Safety, Pathum Thani University, Pathum Thani 12000, Thailand; sittichai.s@ptu.ac.th; 3 Division of Public Health Emergency Management, Office of the Permanent Secretary, Ministry of Public Health, Nonthaburi 11000, Thailand

**Keywords:** primary health care, health service utilization, subdistrict health promoting hospital, expectations, attitudes, perception, rural health, Thailand

## Abstract

**Highlights:**

**What are the main findings?**

- Expectations, attitudes and perception of primary health care service provision were each independently associated with the utilization of subdistrict health promoting hospitals, even after adjustment for sociodemographic characteristics.
- Expectations toward service provision showed the strongest association with utilization, ahead of attitudes and perception.

**What are the implication of the main findings?**

- Improving the utilization of local primary health care services may depend as much on managing patient expectations and communication as on expanding service capacity.
- Subdistrict health promoting hospitals may benefit from targeted outreach on rehabilitation and referral services, which scored lower than other service domains.

**Abstract:**

Background/Objectives: Primary health care (PHC) utilization in rural communities may depend not only on service availability but also on residents’ knowledge of available services, beliefs about service providers, and expectations regarding the value of care. This analytical cross-sectional study examined factors associated with PHC utilization among adults in the catchment areas of subdistrict health promoting hospitals (SHPHs) in Roi Et Province, Northeastern Thailand. Methods: Using multistage sampling, 10 districts, 20 subdistricts, and 20 villages were selected, followed by convenience recruitment of eligible adults within the sampled villages. A total of 461 participants completed a structured questionnaire assessing sociodemographic characteristics, perception, attitudes, expectations, and PHC utilization. Content validity was supported by Index of Item–Objective Congruence values of 0.67 to 1.00, and Cronbach’s alpha coefficients ranged from 0.97 to 0.98. Data were analyzed using descriptive statistics, correlation analysis, and multivariable linear regression. Results: Participants were predominantly female (65.08%), with a mean age of 49.90 years (SD = 15.23). Using predefined equal-width score intervals, perception was classified as moderate (M = 3.59, SD = 0.36), attitudes as positive (M = 3.91, SD = 0.50), expectations as high (M = 4.18, SD = 0.47), and PHC utilization as high (M = 4.13, SD = 0.53). Relatively higher expectation (β = 0.388, *p* < 0.001), attitude (β = 0.279, *p* < 0.001), and perception scores (β = 0.149, *p* < 0.001) were independently associated with greater overall PHC utilization. The adjusted analysis included 446 participants and explained 46.8% of the variance in PHC utilization (R^2^ = 0.468; adjusted R^2^ = 0.453; overall model *p* < 0.001). Conclusions: These findings identify residents’ service-related knowledge, beliefs, and anticipated value as factors associated with PHC utilization and as potential areas for further evaluation in communication and service-improvement initiatives. However, causal direction cannot be established, and the findings should not be generalized beyond the sampled communities in Roi Et Province without further research.

## 1. Introduction

Primary health care (PHC) is widely regarded as the foundation of equitable, accessible, and people-centered health systems. The World Health Organization (WHO) and the United Nations Children’s Fund have emphasized that PHC extends well beyond first-contact clinical care, integrating health promotion, disease prevention, treatment, rehabilitation, and community participation across the life course [1,2]. Strong PHC systems are especially valuable in rural and resource-constrained settings, where they reduce avoidable use of higher-level hospitals, support continuity of care for chronic conditions, and provide a platform for health promotion and emergency response [3,4]. Even so, rural–urban disparities in primary care utilization and resource allocation persist across many health systems, often shaped by socioeconomic status, geographic access, and population-specific healthcare needs [5].

Thailand has achieved broad health insurance coverage through sustained investment in universal health coverage and primary care infrastructure [6,7]. Within this system, subdistrict health promoting hospitals (SHPHs) serve as frontline facilities providing health promotion, disease prevention, basic curative care, rehabilitation, home visits, and referral coordination. These facilities are particularly important in rural provinces, where population aging, a rising burden of chronic disease, and transport barriers increase the need for accessible local health services [8,9]. Recent evidence from Thailand suggests that service quality at SHPHs can vary according to governance and administrative arrangements, underscoring that formal responsibility for care does not, by itself, guarantee consistent service delivery [10]. Consequently, broad population coverage and the physical availability of facilities do not necessarily ensure that residents will use PHC services effectively or appropriately. Utilization may also depend on whether residents understand the services available, trust the providers delivering them, and believe that the services will produce meaningful value in relation to their health needs [11].

Andersen’s behavioral model of health service use proposes that utilization is shaped by predisposing characteristics, enabling resources, need factors, and contextual influences [12,13]. The present study focused on perceptions, attitudes, and expectations because these constructs represent distinct but complementary psychological dimensions through which residents interpret and evaluate PHC services before deciding whether to use them. Perceptions refer to residents’ knowledge and understanding of the scope, availability, functions, and accessibility of services provided by SHPHs. Attitudes refer to residents’ beliefs and evaluative judgments regarding the competence, trustworthiness, safety, fairness, and referral practices of healthcare providers. Expectations refer to residents’ prospective judgments about what the services are likely to deliver and, critically, the value they anticipate receiving, including accessibility, responsiveness, respectful communication, adequate equipment, continuity of care, and confidentiality.

These three constructs were selected because they capture different stages of service appraisal: what residents know about available services, what they believe about the services and providers, and what they predict the services will provide. Perceptions may influence whether residents recognize an SHPH as an appropriate source of care; attitudes may shape their willingness to trust and engage with the facility; and expectations may influence whether the anticipated benefits and value of using the service are considered sufficient. Examining these constructs together may therefore provide a more comprehensive explanation of service utilization than examining general satisfaction or service availability alone. A recent study of health behavior among Thai border communities, for example, found perception and attitude to be significant predisposing factors even after accounting for social support and service access [14]. Evidence from other healthcare settings similarly indicates that the manner in which services are delivered, not merely whether users report general satisfaction, can shape patients’ attitudes and expectations and their subsequent relationships with healthcare providers [15]. Residents who are aware of the services available, hold favorable beliefs about provider competence and trustworthiness, and anticipate that the services will offer sufficient value may be more inclined to consider SHPHs an appropriate point of care rather than bypassing them for district or provincial hospitals [16,17].

Roi Et Province, in Northeastern Thailand, is predominantly rural and agricultural, and its communities face a combination of population aging, a growing burden of noncommunicable diseases, constrained household income, and long travel distances to higher-level facilities [18]. SHPHs consequently play a central role in providing accessible and continuous community-based care, and previous research on diabetes care delivered through SHPHs identified heavy workloads, limited staffing, and insufficiently trained personnel as important constraints on service delivery [19]. Nevertheless, the availability of local facilities alone may not fully explain whether residents choose to use them. Understanding residents’ service-related perceptions, attitudes, and expectations is therefore important for identifying potentially modifiable factors associated with the utilization of rural PHC services. Although previous studies in Thailand and neighboring countries have examined community health volunteers, trust in local healthcare providers, healthcare accessibility, and individual service-related beliefs, evidence on the joint and independent associations of perceptions, attitudes, and expectations with PHC utilization remains limited [20,21]. Few studies have clearly distinguished these three constructs or examined them simultaneously while accounting for sociodemographic characteristics. The present study addresses this gap by evaluating perceptions, attitudes, and expectations as conceptually distinct dimensions of residents’ appraisal of SHPH services. This approach may provide context-specific evidence regarding which dimensions are associated with service utilization in a rural province of Northeastern Thailand.

Because healthcare organization, service capacity, and patterns of utilization may vary across provinces and population groups, the study was designed to characterize adults residing in Roi Et Province rather than to represent all residents of Thailand. Accordingly, this cross-sectional study examined the levels of perceptions, attitudes, expectations, and PHC utilization among adults in Roi Et Province and assessed whether perceptions, attitudes, and expectations toward PHC service provision were associated with the utilization of SHPH services after adjustment for sociodemographic characteristics.

## 2. Materials and Methods

### 2.1. Study Design and Setting

This analytical cross-sectional study was conducted among adults living in Roi Et Province, Northeastern Thailand, and was reported in accordance with the Strengthening the Reporting of Observational Studies in Epidemiology (STROBE) statement [22]. Data were collected from December 2025 to March 2026 using a structured questionnaire administered in community settings within the catchment areas of subdistrict health promoting hospitals (SHPHs).

Roi Et Province is predominantly rural and agricultural and is served by a network of SHPHs that function as first-contact facilities for health promotion, disease prevention, basic medical care, rehabilitation, and referral coordination. The source population comprised adults aged 18 years or older residing in the province, which had a total registered population of 1,059,811 as of December 2024 [23]. Because the study was conducted in selected communities within a single province, the study population was intended to represent the sampled communities rather than all adults in Northeastern Thailand or Thailand as a whole.

### 2.2. Participants and Sampling

The sample size was determined before data collection using the single-population proportion formula, (n = Z^2^p(1 − p)/d^2^). A population proportion of 0.50 was selected because no sufficiently precise prior estimate was available and because this value produces the maximum variance, thereby providing a conservative sample-size estimate. With a two-sided 95% confidence level (Z = 1.96) and a margin of error of 0.05, the estimated minimum sample size was approximately 384 participants. A 20% contingency was added to accommodate incomplete or unusable questionnaires, resulting in a target sample of 461 participants. This calculation was completed before participant recruitment and was used solely to establish the recruitment target.

The original calculation was based on individual-level precision and did not incorporate a design effect for the clustering of participants within villages. It should therefore be interpreted as an individual-level sample-size estimate rather than a cluster-adjusted estimate. Moreover, village identifiers were not retained in the final analytical dataset; consequently, the intraclass correlation coefficient, design effect, and within-village correlation could not be estimated or accounted for in the analysis. These limitations may have affected the precision of the estimated associations and were considered when interpreting the findings.

Eligible participants were Thai adults aged 18 years or older who had resided in Roi Et Province for at least six months, could read and understand Thai, and were able to complete the questionnaire independently. Individuals who were unable to complete the questionnaire because of severe illness or a disability affecting communication, as well as those who declined to participate, were excluded. Only participants with complete data for all variables included in the multivariable model were included in the adjusted regression analysis. All 461 participants were included in the descriptive analyses, whereas 446 participants with complete covariate data were included in the multivariable regression analysis.

A multistage area-selection procedure was used to identify the study locations, followed by non-probability recruitment of individual participants within the selected villages. First, 10 of the 20 districts in Roi Et Province were selected by simple random sampling. Second, two subdistricts were selected from each sampled district by systematic sampling, resulting in 20 subdistricts. Third, one village was selected from each sampled subdistrict by systematic sampling, resulting in 20 selected villages. At the individual-participant stage, village health volunteers and local coordinators assisted in informing potentially eligible residents about the study. Eligible adults who were present or accessible during the data-collection period and who voluntarily agreed to participate were recruited until the predetermined target was approached.

Although districts, subdistricts, and villages were selected using probability-based procedures, the final recruitment of individuals was based on availability and voluntary participation and was therefore non-probabilistic. Accordingly, the study sample should not be considered a probability sample of all adults in Roi Et Province. The potential for selection bias and the resulting limitations on population-level generalizability were acknowledged when interpreting the results.

### 2.3. Study Variables and Measurement

The questionnaire comprised five parts.

- Part 1 collected sociodemographic and health-related information, including gender, age, marital status, educational level, average monthly income, occupation, chronic conditions, and duration of health service utilization at SHPHs.
- Part 2 assessed perceptions of primary health care (PHC) service provision, using 16 items across four domains: basic healthcare services, community health promotion, disease prevention and control in the community, and post-illness rehabilitation. In this study, perceptions represented participants’ knowledge and understanding of the scope, availability, and functions of services provided by SHPHs.
- Part 3 assessed attitudes toward PHC service provision using 16 items across four domains: health promotion and disease prevention, the safety of basic medical care services, healthcare personnel performance, and referral systems for conditions beyond facility capacity. Attitudes represented participants’ beliefs and evaluative judgments regarding the competence, trustworthiness, safety, fairness, and performance of SHPH services and personnel.
- Part 4 assessed expectations toward PHC service provision using 16 items across four domains: PHC service delivery, healthcare personnel, medical equipment and supplies, and communication and service accessibility. Expectations represented participants’ prospective judgments regarding what SHPH services were likely to provide and the value they anticipated receiving from those services.
- Part 5 assessed self-reported PHC utilization at SHPHs using 16 items across four service domains: health promotion services, disease prevention and control services, basic medical care services, and rehabilitation services. Utilization was assessed separately for the four service domains. A domain score was calculated by averaging the items within each domain, and an overall PHC utilization score was calculated by averaging all 16 utilization items. Thus, the primary dependent variable represented the combined level of self-reported utilization across the four categories of PHC services. Domain-specific utilization scores were retained for additional descriptive and exploratory analyses because the associations of perceptions, attitudes, and expectations could differ according to service type.

Items in Parts 2–5 used five-point Likert-type response scales appropriate to each construct. For each scale, domain scores and the overall score were calculated as arithmetic means, resulting in possible scores ranging from 1.00 to 5.00. Higher scores indicated more favorable perceptions, attitudes, and expectations or greater self-reported utilization.

For descriptive interpretation, the possible score range was divided into three equal-width intervals using the formula ((5 − 1)/3 = 1.33). Perception, expectation, and utilization scores were categorized as low (1.00–2.33), moderate (2.34–3.66), or high (3.67–5.00). Attitude scores were categorized as negative (1.00–2.33), neutral (2.34–3.66), or positive (3.67–5.00). These categories were used only to describe score levels and were not treated as validated clinical thresholds. All correlation and regression analyses used the original continuous scores rather than the three-category classifications.

### 2.4. Content Validity and Reliability

The questionnaire was developed from the relevant literature, the study’s conceptual framework, and the operational definitions of the study constructs. All instrument development, expert review, and pilot testing were completed before the main study data collection, which commenced on 25 December 2025.

Content validity was evaluated independently by three experts in behavioral science, public health, and research methodology. Each item was rated as −1 (not congruent), 0 (uncertain), or +1 (congruent), and the item-level Index of Item–Objective Congruence (IOC) was calculated by averaging the three expert ratings. An IOC value of ≥0.50 was considered acceptable. The instrument comprised four 16-item scales assessing perception, attitude, expectation, and primary health care utilization. Across the four scales, item-level IOC values ranged from 0.67 to 1.00. Six items were revised for wording and clarity following expert review, and no items were removed before pilot testing. The IOC findings were interpreted specifically as evidence of item–objective congruence based on expert judgment and not as evidence of construct or factorial validity.

The revised questionnaire was pilot-tested among 40 adults in Maha Sarakham Province who had characteristics similar to those of the target population but were not included in the main study. The pilot test assessed clarity, comprehensibility, administration procedures, and internal consistency. Participants reported no major difficulties in understanding or completing the questionnaire; therefore, no further revisions were made after pilot testing.

Internal consistency was assessed separately for each scale using the pilot-test data. Cronbach’s alpha coefficients were 0.97 for perception, 0.97 for attitude, 0.98 for expectation, and 0.98 for primary health care utilization. An overall alpha coefficient across all 64 items was not calculated because the questionnaire was designed to measure four conceptually distinct constructs. Given that the scale-specific alpha coefficients exceeded 0.95, additional item-level reliability diagnostics were examined using the original pilot-test dataset. Corrected item–total correlations ranged from 0.58 to 0.86, inter-item correlations ranged from 0.31 to 0.79, and Cronbach’s alpha if item deleted ranged from 0.96 to 0.98. Although some inter-item correlations were relatively high, review of the item content did not identify items that were judged to be conceptually identical, and deletion of individual items did not materially improve internal consistency. Accordingly, all items were retained to preserve coverage of the prespecified content domains.

The high Cronbach’s alpha coefficients were therefore interpreted cautiously because they may partly reflect similarity among items and do not, by themselves, establish unidimensionality or construct validity. The psychometric evaluation in this study was limited to expert-assessed item–objective congruence and pilot-sample internal consistency; factorial validity and test–retest reliability were not assessed. Further psychometric evaluation in independent samples is warranted.

### 2.5. Data Collection

Trained data collectors explained the study objectives, procedures, potential risks, and participant rights to eligible adults. Those who agreed to participate provided written informed consent before completing the questionnaire. Completed questionnaires were checked for completeness before data entry, and no personally identifiable information was recorded.

Data collectors followed standardized instructions and were trained not to influence participants’ responses. Participants completed the questionnaire independently in a private or suitably separated area whenever possible to reduce social desirability and interviewer-related bias.

### 2.6. Statistical Analysis

Data were analyzed using IBM SPSS Statistics, version 26.0 (IBM Corp., Armonk, NY, USA) [24], with selected analyses verified using Python, version 3.12 (Python Software Foundation, Wilmington, DE, USA). Categorical variables were summarized as frequencies and percentages, whereas continuous variables were summarized as means and standard deviations (SDs) or medians and interquartile ranges (IQRs), as appropriate. Bivariate associations among sociodemographic characteristics, duration of subdistrict health promoting hospital (SHPH) use, perception, attitude, expectation, and overall primary health care (PHC) utilization were examined using Pearson or Spearman correlation coefficients.

Multivariable linear regression was used to estimate the adjusted associations of perception, attitude, and expectation scores with overall PHC utilization. Covariates selected a priori included gender, age, marital status, educational level, monthly income, farming status, chronic conditions, and duration of SHPH use. Occupation was coded as farming versus nonfarming, income was analyzed per 1000 Thai Baht (THB), and categorical variables were indicator coded. The enter method was used for the primary model, while backward elimination was conducted as a sensitivity analysis.

Regression assumptions were assessed using residual diagnostics, variance inflation factors, and measures of influential observations. Because heteroscedasticity was detected, heteroscedasticity-consistent (HC3) robust standard errors were used. Village-level clustering could not be directly accounted for because village identifiers were not retained in the analytical dataset. Exploratory adjusted models were also fitted separately for the four PHC utilization domains. Results are reported as unstandardized coefficients (B), standardized coefficients (β), 95% confidence intervals (CIs), and two-sided *p*-values, with statistical significance set at *p* < 0.05.

### 2.7. Ethical Considerations

The study was conducted in accordance with the Declaration of Helsinki and approved by the Human Research Ethics Committee of Suan Sunandha Rajabhat University (COE No. 2-583/2025; approval date: 24 December 2025). Participation was voluntary, and participants could withdraw at any time without consequence.

## 3. Results

### 3.1. Participant Characteristics

All 461 participants were included in the descriptive analyses. Most participants were female (n = 300, 65.08%), married (n = 293, 63.56%), and educated to the primary-school level (n = 152, 32.97%). The mean age was 49.90 years (SD = 15.23). Farmers constituted the largest occupational group (n = 181, 39.26%), and 344 participants (74.62%) reported no chronic conditions. The median duration of subdistrict health promoting hospital (SHPH) service use was 19 years (IQR = 9–28; range = 2–49). Among the 446 participants with available income data, the median monthly income was 8000 Thai Baht (THB) (IQR = 5000–10,000; range = 2000–26,500) (Table 1).

### 3.2. Perception, Attitudes, Expectations and PHC Utilization

Using the predefined equal-width score intervals, the overall perception score was classified as moderate (M = 3.59, SD = 0.36). Community health promotion had the highest perception-domain score (M = 3.63, SD = 0.60), whereas post-illness rehabilitation had the lowest score (M = 3.51, SD = 0.60). The overall attitude score was classified as positive (M = 3.91, SD = 0.50), with healthcare personnel performance receiving the highest domain score (M = 4.10, SD = 0.68). The overall expectation score was classified as high (M = 4.18, SD = 0.47), and the healthcare personnel domain had the highest score (M = 4.21, SD = 0.55). Overall primary health care (PHC) utilization at SHPHs was classified as high (M = 4.13, SD = 0.53). Among the four service domains, utilization was highest for disease prevention and control services (M = 4.23, SD = 0.66) and lowest for rehabilitation services (M = 3.96, SD = 0.76) (Table 2).

### 3.3. Bivariate Associations with PHC Utilization

In the income-complete sample (n = 446), duration of SHPH service use (r = 0.244, *p* < 0.001), perception (r = 0.354, *p* < 0.001), attitude (r = 0.550, *p* < 0.001), expectation (r = 0.586, *p* < 0.001), and married status (r = 0.142, *p* = 0.003) were positively correlated with PHC utilization. Farming status was not significantly correlated with utilization (r = 0.089, *p* = 0.060). (Table 3).

### 3.4. Multivariable Regression Analysis

In the adjusted linear regression model, expectation, attitude, and perception remained positively associated with PHC utilization after adjustment for gender, age, marital status, education level, income, farming status, chronic conditions, and duration of health service utilization. Expectation had the largest standardized association (B = 0.437, SE = 0.063, β = 0.388, 95% CI: 0.313 to 0.560, *p* < 0.001), followed by attitude (B = 0.298, SE = 0.060, β = 0.279, 95% CI: 0.181 to 0.415, *p* < 0.001) and perception (B = 0.221, SE = 0.060, β = 0.149, 95% CI: 0.103 to 0.338, *p* < 0.001). Married participants (vs. single) reported modestly higher utilization than single participants (B = 0.099, SE = 0.048, β = 0.089, 95% CI: 0.005 to 0.194, *p* = 0.040); farming status was not significant at the conventional threshold (B = 0.093, SE = 0.048, β = 0.085, 95% CI: −0.001 to 0.188, *p* = 0.053), and this borderline association was not stable across sensitivity analyses (Section 4). The adjusted model explained 46.81% of the variance in PHC utilization (R^2^ = 0.4681; adjusted R^2^ = 0.4533; F(12, 433) = 33.8381, *p* < 0.001; SE of the estimate = 0.3971). Variance inflation factor values ranged from 1.046 to 2.401, indicating no problematic multicollinearity. Because heteroscedasticity was detected, HC3 robust standard errors were used. Village-level clustering could not be modeled because village identifiers were unavailable in the analytical dataset; therefore, the reported standard errors and confidence intervals do not account for potential within-village correlation. (Table 4).

## 4. Discussion

This study examined the psychosocial and sociodemographic factors associated with self-reported primary health care (PHC) utilization at subdistrict health promoting hospitals (SHPHs) among adults in Roi Et Province, Northeastern Thailand. Based on the predefined score-classification criteria, overall PHC utilization was categorized as high, with the highest score observed for disease prevention and control services and the lowest score observed for rehabilitation services. Relatively higher expectation, attitude, and perception scores were independently associated with greater overall PHC utilization, with expectations showing the largest standardized association. These findings indicate that residents’ knowledge of available services, beliefs about service providers, and anticipated value of care were associated with their reported utilization of SHPH services. However, the cross-sectional design does not establish the temporal direction or causality of these relationships.

The high overall utilization score should be interpreted as a descriptive classification based on the study’s predefined scoring intervals rather than as an objective measure of the number of healthcare visits. Nevertheless, the observed pattern is consistent with the central role of SHPHs in delivering health promotion, disease prevention, basic medical care, and community outreach in rural Thailand. Disease prevention and control services received the highest utilization score, which may reflect the long-standing delivery of screening, vaccination, health education, and disease-control activities through local facilities and community-based networks. Recent evidence has similarly highlighted the contribution of village health volunteers to outreach, health communication, and continuity of community care in rural Thailand [20]. Rehabilitation services received the lowest utilization score, although the score remained within the predefined high category. This relative difference may indicate lower awareness, availability, perceived relevance, or accessibility of rehabilitation and longer-term recovery services compared with preventive and basic medical services. Evidence from resource-constrained primary care settings has identified limited public awareness of the scope and potential benefits of rehabilitation as an important barrier to service uptake [27]. Further service-specific research is required to determine whether this pattern reflects differences in community knowledge, service capacity, referral practices, or actual need.

Relatively higher expectation scores showed the strongest adjusted association with overall PHC utilization. This finding does not mean that “high expectations” caused greater utilization; rather, participants who reported higher expectation scores relative to others also tended to report greater use of SHPH services. Expectations in this study represented prospective judgments about the accessibility, responsiveness, communication, equipment, confidentiality, and anticipated value of PHC services. The finding is consistent with expectation disconfirmation theory, which proposes that the relationship between anticipated and experienced service performance contributes to users’ evaluations and subsequent service decisions [17]. Research in healthcare settings has also shown that patients’ evaluations depend not only on technical service quality but also on provider behavior and the way care is delivered [15]. Recent evidence from other primary care settings similarly shows that gaps between patients’ expectations and perceived service quality shape their evaluations of care [28]. The observed association may therefore reflect that residents who anticipated greater value, responsiveness, and appropriateness from SHPH services were also more likely to regard these facilities as acceptable sources of care. Reverse directionality is also possible, however, because repeated service use may shape or reinforce expectations over time.

Relatively higher attitude scores were also associated with greater PHC utilization. Attitudes represented residents’ beliefs and evaluations concerning provider competence, service safety, fairness, personnel performance, and the reliability of referral processes. This relationship is consistent with behavioral theory, in which favorable evaluations of a service are associated with stronger intentions to use it when the service is viewed as useful, safe, and socially acceptable [16]. Recent research in Thai border communities similarly identified attitudes and perceptions as relevant predisposing factors for health-related behavior after consideration of social support and service accessibility [14]. In the context of rural SHPHs, the association may reflect the importance of trust, respectful interactions, confidence in healthcare personnel, and perceived consistency of care. However, the present findings cannot determine whether favorable attitudes preceded service utilization or developed through previous positive experiences with SHPH services.

Perception scores remained independently associated with overall PHC utilization, although their standardized association was smaller than those of expectations and attitudes. In this study, perceptions referred primarily to residents’ knowledge and understanding of the scope, availability, and functions of SHPH services. The overall perception score was classified as moderate, suggesting that some participants may not have been fully aware of the range of services available, particularly rehabilitation, follow-up care, and referral coordination. This interpretation is consistent with the broader understanding that access depends not only on the formal availability of healthcare but also on whether individuals recognize that a service exists, understand its purpose, and believe that they can use it [11]. In Thailand, differences in governance, staffing, resources, and administrative arrangements may also affect the consistency of services delivered by SHPHs and consequently shape community perceptions of local facilities [10]. Recent regional studies have additionally linked limited health literacy with poorer awareness, healthcare decision-making, and appropriate use of medicines and health services among older adults [29,30]. Communication strategies may therefore need to describe the specific functions of SHPHs rather than simply encouraging residents to attend the facilities.

The descriptive findings also suggest that PHC utilization was not uniform across service categories. Exploratory domain-specific models indicated that the role of the psychological constructs differed by service type (Table 5). For health promotion services, only expectation was independently associated with utilization (β = 0.387, *p* < 0.001); perception and attitude were not significant. For disease prevention and control, basic medical care, and rehabilitation services, all three constructs were independently associated with utilization, with attitude showing the strongest association for basic medical care specifically (β = 0.332), exceeding expectation (β = 0.270) in that domain alone. Explanatory power was also higher for the two curative/preventive domains (adjusted R^2^ = 38.36–40.03%) than for health promotion and rehabilitation (adjusted R^2^ = 22.35–24.51%), suggesting other unmeasured factors play a larger role in those two domains. These service-specific findings should be interpreted as exploratory and confirmed in future studies designed to compare utilization across distinct types of PHC services.

The revised regression approach represented occupation as farming versus nonfarming and did not include separate occupation categories or unemployment status because of their potential overlap with education and income. This revised model did not identify a statistically significant independent association for farming status (*p* = 0.053) or for age, education, income, or chronic condition at the conventional threshold. The association between farming status and utilization was, moreover, not stable across specifications: it strengthened under backward elimination (*p* = 0.005) but was attenuated to non-significance when three influential cases were excluded (*p* = 0.196), and is therefore not interpreted as a reliable finding. Married participants (vs. single) reported modestly higher utilization than single participants (*p* = 0.040–0.041 across sensitivity analyses); because this association was not significant in the original, pre-respecification model, it is presented as hypothesis-generating rather than a confirmed effect. Any sociodemographic association identified after reanalysis should be interpreted cautiously because occupational, marital, and economic circumstances may reflect heterogeneous differences in financial resources, health needs, transportation, social support, and connection with community health networks [30,31]. A comparable study using the same three constructs at subdistrict health promoting hospitals elsewhere in Thailand explained substantially less variance in utilization (adjusted R^2^ = 28.7%) [32], which may reflect differences in facility transition status, sampling, or covariate adjustment between settings, and suggests the strength of these associations is not uniform across provinces.

### 4.1. Implications for PHC Policy and Practice

The findings identify several areas that may be considered for further evaluation in PHC practice rather than demonstrating that changes in these areas will necessarily increase utilization. First, SHPHs could assess whether residents clearly understand the scope of available services, particularly rehabilitation, follow-up care, and referral coordination. Service information should be communicated in plain language and through channels accessible to rural populations. Second, facilities could evaluate whether information about operating procedures, waiting times, referral pathways, equipment and supply availability, confidentiality, and communication channels is sufficiently clear to support realistic expectations. Third, continued attention to respectful communication, privacy, equitable treatment, and perceived provider competence may help maintain favorable attitudes toward local services. Finally, village health volunteers may assist in identifying misunderstandings about service scope and communicating accurate information to households that have limited contact with formal health services [20]. Because the study was cross-sectional, the effectiveness of these approaches should be tested through longitudinal or intervention-based research before they are adopted as strategies for increasing PHC utilization.

### 4.2. Limitations

This study has several limitations. Its cross-sectional design precludes conclusions regarding temporality or causality. The use of a single self-reported questionnaire may have introduced recall, social desirability, and common method biases. Although multistage sampling was used, participants were recruited nonprobabilistically at the final stage, and the sample-size calculation did not incorporate a design effect. Village identifiers were unavailable, preventing adjustment for within-village correlation; HC3 standard errors addressed heteroscedasticity but not clustering. The study was conducted in selected communities within one province, limiting generalizability. Missing income data reduced the adjusted-analysis sample, and the factor structure and construct validity of the instruments were not evaluated.

## 5. Conclusions

Among adults in the sampled communities of Roi Et Province, overall primary health care (PHC) utilization at subdistrict health promoting hospitals (SHPHs) was classified as high, with the highest utilization score for disease prevention and control services and the lowest for rehabilitation services. Perception of PHC services was moderate, whereas attitudes were positive and expectations were high. Relatively higher expectation, attitude, and perception scores were independently associated with greater PHC utilization, with expectations showing the largest standardized association. These findings identify service-related knowledge, beliefs, and anticipated value as potential areas for further evaluation in PHC communication and service improvement. However, the cross-sectional design precludes causal inference, and the findings should not be generalized beyond the sampled communities. Longitudinal and intervention-based studies are needed to determine whether modifying these factors influences service utilization.

## Figures and Tables

**Table 1:** Sociodemographic characteristics of participants (n = 461).

Characteristic	n	%
Gender		
Male	161	34.92
Female	300	65.08
Age, years, mean (SD)	49.90 (15.23)	
Age, median (IQR), range	52 (38–61), 19–87	
Marital status		
Single	119	25.81
Married	293	63.56
Widowed/divorced/separated	49	10.63
Education level		
No formal education	8	1.74
Primary education	152	32.97
Lower secondary education	99	21.48
Upper secondary/vocational certificate	102	22.13
Diploma/higher vocational certificate	31	6.72
Bachelor’s degree	67	14.53
Master’s degree or higher	2	0.43
Occupation		
Farmer	181	39.26
General laborer	110	23.86
Business owner/self-employed	47	10.20
Unemployed	39	8.46
Government officer/state enterprise employee	33	7.16
Student	27	5.86
Private/company employee	24	5.21
Chronic conditions		
No	344	74.62
Yes	117	25.38
Duration of SHPH utilization, years, mean (SD)	18.36 (11.14)	
Duration, median (IQR), range	19 (9–28), 2–49	
Average monthly income, THB, mean (SD)	8939.63 (4843.71)	
Income, median (IQR), range	8000 (5000–10,000), 2000–26,500	

THB = Thai Baht; IQR = interquartile range; SD = standard deviation; SHPH = subdistrict health promoting hospital.

**Table 2:** Perception, attitudes, expectations and primary health care utilization (n = 461).

Variable/Domain	M	SD	Level
Perception of PHC service provision	3.59	0.36	Moderate
Basic health care services	3.61	0.55	Moderate
Community health promotion	3.63	0.60	Moderate
Disease prevention and control in the community	3.62	0.58	Moderate
Post-illness rehabilitation	3.51	0.60	Moderate
Attitudes toward PHC service provision	3.91	0.50	Positive
Health promotion and disease prevention	3.81	0.70	Positive
Safety of basic medical care services	3.97	0.67	Positive
Healthcare personnel performance	4.10	0.68	Positive
Referral systems beyond facility capacity	3.76	0.62	Positive
Expectations toward PHC service provision	4.18	0.47	High
PHC service delivery	4.13	0.59	High
Healthcare personnel	4.21	0.55	High
Medical equipment and supplies	4.19	0.58	High
Communication and service accessibility	4.19	0.60	High
PHC utilization in SHPHs	4.13	0.53	High
Health promotion services	4.21	0.56	High
Disease prevention and control services	4.23	0.66	High
Basic medical care services	4.10	0.67	High
Rehabilitation services	3.96	0.76	High

M = mean; SD = standard deviation; PHC = primary health care; SHPHs = subdistrict health promoting hospitals. Score levels were derived by dividing the possible range (1.00–5.00) into three equal-width class intervals of 1.33 points each, following common practice for descriptively interpreting Likert-type mean scores [25,26]. These bands are descriptive only and were not used as validated clinical or diagnostic thresholds; all correlation and regression analyses used the continuous scores.

**Table 3:** Pearson correlation matrix for all study variables (n = 446, income-complete subsample).

1. Female	1	2	3	4	5	6	7	8	9	10	11	12
2. Age	−0.115											
3. Married	−0.016	0.384										
4. Widowed/divorced/separated	−0.075	0.285	−0.448									
5. Education level	0.123	−0.606	−0.301	−0.128								
6. Income (per 1000 THB)	−0.033	−0.259	−0.054	−0.124	0.471							
7. Farming (yes vs. no)	0.029	0.390	0.264	0.128	−0.371	−0.209						
8. Chronic condition	−0.095	0.333	0.090	0.196	−0.284	−0.115	0.184					
9. Duration of SHPH service use	0.002	0.252	0.182	−0.020	−0.133	0.020	0.081	0.117				
10. Perception	−0.027	0.054	0.031	0.044	−0.075	−0.014	−0.033	−0.092	0.133			
11. Attitude	0.008	−0.004	0.049	−0.067	−0.018	0.026	−0.047	−0.011	0.240	0.405		
12. Expectation	0.006	0.005	0.096	−0.044	−0.051	0.031	0.021	0.094	0.230	0.222	0.511	
13. PHC utilization	0.058	0.045	0.142	−0.042	−0.069	0.014	0.089	0.031	0.244	0.354	0.550	0.586

Variables: 1 = Female; 2 = Age; 3 = Married; 4 = Widowed/divorced/separated; 5 = Education level; 6 = Income (per 1000 THB); 7 = Farming (yes vs. no); 8 = Chronic condition; 9 = Duration of SHPH service use; 10 = Perception; 11 = Attitude; 12 = Expectation; 13 = PHC utilization. The largest inter-predictor correlation was between age and education (r = −0.606); corresponding VIF values (2.401 and 2.032) remained below conventional thresholds for concern.

**Table 4:** Adjusted linear regression analysis of factors associated with primary health care utilization (n = 446).

Variable	B	SE	β	*t*	95% CI for B	*p*
Constant	0.237	0.279	–	0.850	−0.311 to 0.784	0.396
Female (vs. male)	0.062	0.040	0.055	1.547	−0.017 to 0.141	0.123
Age, years	−0.002	0.002	−0.050	−0.944	−0.005 to 0.002	0.346
Married (vs. single)	0.099	0.048	0.089	2.063	0.005 to 0.194	0.040
Widowed/divorced/separated (vs. single)	0.065	0.089	0.037	0.732	−0.109 to 0.240	0.464
Education level	−0.003	0.019	−0.009	−0.175	−0.040 to 0.033	0.861
Income per 1000 THB	0.002	0.004	0.015	0.378	−0.007 to 0.011	0.706
Farming (vs. non-farming)	0.093	0.048	0.085	1.941	−0.001 to 0.188	0.053
Chronic condition (yes vs. no)	−0.006	0.050	−0.005	−0.116	−0.103 to 0.092	0.908
Duration of service utilization, years	0.003	0.002	0.058	1.570	−0.001 to 0.006	0.117
Perception of PHC service provision	0.221	0.060	0.149	3.703	0.103 to 0.338	<0.001
Attitudes toward PHC service provision	0.298	0.060	0.279	4.987	0.181 to 0.415	<0.001
Expectations toward PHC service provision	0.437	0.063	0.388	6.953	0.313 to 0.560	<0.001

Model: R = 0.684, R^2^ = 0.4681 (46.81%), adjusted R^2^ = 0.4533 (45.33%), SE of the estimate = 0.397, F(12, 433) = 33.8381, *p* < 0.001. B = unstandardized coefficient; β = standardized coefficient; CI = confidence interval; SE = standard error; VIF = variance inflation factor; THB = Thai Baht. Reference categories: male (gender), single (marital status), non-farming (occupation), no chronic condition. Standard errors are heteroscedasticity-consistent (HC3) robust standard errors. They account for heteroscedasticity but do not account for potential within-village correlation because village identifiers were unavailable in the analytical dataset.

**Table 5:** Adjusted regression coefficients for perception, attitude, and expectation associated with primary health care utilization, by service domain (n = 446).

Domain (DV)	Adj. R^2^	Perception B (β), *p*	Attitude B (β), *p*	Expectation B (β), *p*
Health promotion services	22.35%	0.021 (0.013), 0.768	0.052 (0.046), 0.434	0.456 (0.387), <0.001
Disease prevention and control services	38.36%	0.274 (0.149), 0.001	0.319 (0.240), <0.001	0.543 (0.388), <0.001
Basic medical care services	40.03%	0.323 (0.176), <0.001	0.441 (0.332), <0.001	0.378 (0.270), <0.001
Rehabilitation services	24.51%	0.265 (0.126), 0.010	0.381 (0.251), <0.001	0.369 (0.231), <0.001

Exploratory analysis; full covariate set as in Table 4 (gender, age, marital status, education, income, farming status, chronic condition, duration of service use, perception, attitude, expectation); F(12, 433) ranged from 10.9005 to 32.5090, all *p* < 0.001.

## Data Availability

The data presented in this study are not publicly available because of ethical and institutional restrictions related to participant confidentiality. Deidentified data may be made available from the corresponding authors upon reasonable request and with approval from the Research and Development Institute, Suan Sunandha Rajabhat University.

## Abbreviations

The following abbreviations are used in this manuscript:

Abbreviation	Definition
adjusted R^2^	Adjusted coefficient of determination
B	Unstandardized Coefficients
β	Standardized regression coefficient
CI	Confidence interval
COE	Certificate of Ethical Approval
95% CI	95.00% Confidence Interval for B
F	Fisher’s F-statistic
GenAI	Generative Artificial Intelligence
IOC	Index of Item-Objective Congruence
IQR	Interquartile range
M	Mean
N	Population Size
n	Sample Size
PHC	Primary health care
p	Probability value
R	Multiple correlation coefficient
R^2^	Coefficient of determination
r	Pearson correlation coefficient
SD	Standard deviation
SE	Standard error
SPSS	Statistical Package for the Social Sciences
SHPH, SHPHs	Subdistrict health promoting hospital
STROBE	Strengthening the Reporting of Observational Studies in Epidemiology
THB	Thai baht
*t*	Student’s *t*-statistic
UNICEF	United Nations Children’s Fund
VIF	Variance inflation factor
WHO	World Health Organization
Y	PHC utilization in SHPHs, the dependent variable
%	Percentage
